# Rationale and design of the SENTRY trial: sentinel node and organ-preserving surgery *versus* segmental resection in stage I colon cancer

**DOI:** 10.1093/bjsopen/zrag075

**Published:** 2026-07-21

**Authors:** Bart C T van de Laar, Daan J Sikkenk, Pieter J Tanis, Paul M Verheijen, Matthijs P Schwartz, Halil Akol, René Arensman, Frank J Voskens, Leon M G Moons, Wouter H de Vos tot Nederveen Cappel, Henderik L van Westreenen, Wouter B Nagengast, Esther C J Consten

**Affiliations:** Department of Surgery, University of Groningen, University Medical Center Groningen, Groningen, the Netherlands; Department of Surgery, Meander Medical Center, Amersfoort, the Netherlands; Department of Surgery, University of Groningen, University Medical Center Groningen, Groningen, the Netherlands; Department of Surgery, Meander Medical Center, Amersfoort, the Netherlands; Department of Oncologic and Gastrointestinal Surgery, Erasmus MC Cancer Institute, Rotterdam, the Netherlands; Department of Surgery, Meander Medical Center, Amersfoort, the Netherlands; Department of Gastroenterology and Hepatology, Meander Medical Center, Amersfoort, the Netherlands; Department of Gastroenterology and Hepatology, Meander Medical Center, Amersfoort, the Netherlands; Department of Pathology, Meander Medical Center, Amersfoort, the Netherlands; Department of Surgery, Meander Medical Center, Amersfoort, the Netherlands; Department of Gastroenterology and Hepatology, University Medical Center Utrecht, Utrecht, the Netherlands; Department of Gastroenterology and Hepatology, Isala, Zwolle, the Netherlands; Department of Surgery, Isala, Zwolle, the Netherlands; Department of Gastroenterology and Hepatology, University of Groningen, University Medical Center Groningen, Groningen, the Netherlands; Department of Surgery, University of Groningen, University Medical Center Groningen, Groningen, the Netherlands; Department of Surgery, Meander Medical Center, Amersfoort, the Netherlands

**Keywords:** colonoscopy-assisted laparoscopic wedge resection, fluorescence-guided surgery, indocyanine green, sentinel lymph node biopsy, near-infrared fluorescence, colorectal cancer

Population screening has increased local resections of early-stage colon cancer (CC). Current Dutch guidelines use histopathological risk stratification after local resection of p (pathological) T1–T2 CC based on factors including poor differentiation, lymphovascular invasion, high-grade tumour budding, and Haggitt level 4. The estimated risk of lymph node metastases (LNM) further depends on pT stage, polyp morphology, and margin status. Patients with a higher risk of LNM often undergo additional surgery with segmental resection. Many of these patients ultimately have no LNM and may therefore be exposed to postoperative morbidity, mortality, and impaired bowel function without oncological benefit.

Sentinel lymph node (SLN) biopsy using indocyanine green (ICG) may offer nodal staging while preserving the colon. In early-stage CC, SLN mapping with ICG has shown approximately 80% sensitivity^1^. Colonoscopy-assisted laparoscopic wedge resection (CAL-WR)^2^ can be combined with intraoperative ICG injection, allowing local resection and SLN biopsy in a single procedure.

SENTRY is a prospective, multicentre, non-inferiority, partially randomized patient preference trial (RPPT) conducted in the Netherlands (Registration number: NCT06652672; http://www.clinicaltrials.gov; see *Supplementary material* for full trial protocol). Patients with pT1–T2 CC after endoscopic resection and an LNM risk > 15% or those with a macroscopically suspected T1 tumour unsuitable for endoscopic resection are eligible for inclusion. Patients either choose between treatments or consent to 1 : 1 randomization between organ-preserving surgery and standard segmental resection. An RPPT design was chosen because strong treatment preferences were anticipated, which could have limited recruitment in a conventional randomized trial. This design may improve accrual and external validity^3^.

In the organ-preserving arm, intraoperative colonoscopy is used to inject ICG submucosally in four quadrants around the tumour or endoscopic resection scar. Up to four SLNs are identified with near-infrared fluorescence imaging and excised for ultrastaging^4^. CAL-WR is then performed to remove the lesion (*Fig. 1*). Patients with negative SLNs, defined as no metastasis or isolated tumour cells only, which are not considered prognostically relevant^4^, undergo intensive follow-up with carcinoembryonic antigen testing, colonoscopies, and repeated computed tomography imaging for early recurrence detection. If no SLN is identified or if CAL-WR is technically unfeasible, conversion to segmental resection is performed. Completion segmental resection is recommended in the event of LNM > 0.2 mm, pT3–T4 disease, or incomplete local resection. The comparator arm undergoes standard segmental resection with adjuvant therapy and follow-up in accordance with Dutch guidelines^5^.

**Fig. 1:**
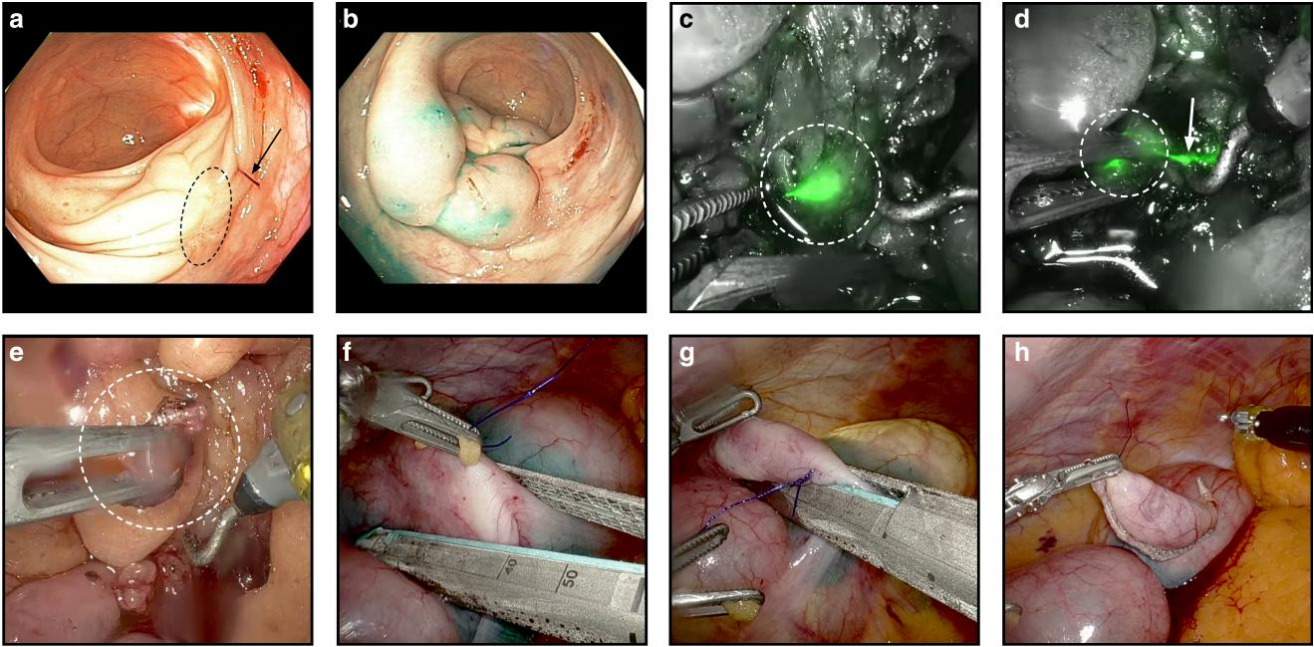
Endoscopic and surgical steps in CAL-WR and SLN biopsy. **a** Endoscopic view of the polypectomy scar (dashed ellipse); the suture used for traction during CAL-WR is visible adjacent to the scar (arrow). **b** The four submucosal blebs after indocyanine green injection. **c** Near-infrared fluorescence view on the da Vinci Xi system (Intuitive Surgical, Sunnyvale, CA, USA) showing an SLN (dashed circle). **d** An SLN (dashed circle) held with Cadiere forceps, with a lymphatic vessel draining into the node (arrow). **e** Excised SLN under white-light view (dashed circle). **f** CAL-WR with traction applied to the suture. **g** Stapler positioning. **h** Completed resection. CAL-WR, colonoscopy-assisted laparoscopic wedge resection; SLN, sentinel lymph node.

The trial has a sequential design. The feasibility phase includes the first 40 patients in the organ-preserving arm and will proceed only to the main phase if the SLN detection rate is > 80%. The main phase evaluates non-inferiority for 3-year overall recurrence. Secondary outcomes include 3- and 5-year locoregional and distant recurrence, overall survival, technical success, complications, postoperative mortality, patient preferences, quality of life, and costs.

Sample size calculations are based on an estimated 3-year recurrence rate of 3% after standard segmental resection for stage I CC. Given an 80% sensitivity of SLN biopsy^1^ and an estimated 20% rate of LNM, recurrence risk may be increased. An 8% non-inferiority margin was considered acceptable and increased to 11% to account for the approximately 3% perioperative mortality associated with segmental resection, as well as the expected lower morbidity and mortality of organ-preserving surgery. In addition, increasing evidence suggests that locoregional recurrences can often be successfully treated with salvage surgery without substantially affecting distant metastasis or cancer-specific survival^6^. With this margin, 119 patients are required per group. After accounting for technical failure, completion surgery, and loss to follow-up, a minimum of 179 and 162 patients will be included in the intervention and comparator groups, respectively.

Initiated after ethics approval, the SENTRY trial will determine whether CAL-WR combined with SLN biopsy can safely reduce segmental resections in select patients with early-stage CC. If non-inferior outcomes are demonstrated, this approach may offer an organ-preserving alternative.

## Data Availability

Data can be shared upon reasonable request from the corresponding author.
